# The role of gut microbiota and amino metabolism in the effects of improvement of islet β-cell function after modified jejunoileal bypass

**DOI:** 10.1038/s41598-021-84355-x

**Published:** 2021-02-26

**Authors:** Cai Tan, Zhihua Zheng, Xiaogang Wan, Jiaqing Cao, Ran Wei, Jinyuan Duan

**Affiliations:** 1 Department of Women’s Health, Jiangxi Maternal and Child Health Hospital, Nanchang, 330006 China; 2 Department of General Surgery, The Second Affiliated Hospital of Nanchang University, Nanchang, 330006 China; 3 Department of Gastrointestinal Surgery, The Second Affiliated Hospital of Nanchang University, Nanchang, 330006 China; 4 Department of Colorectal Surgery, National Cancer Center/National Clinical Research Center for Cancer/Cancer Hospital, Chinese Academy of Medical Sciences, Peking Union Medical College, Beijing, 100021 China; 5 Department of General Surgery, The First Affiliated Hospital of Nanchang University, Gastrointestinal Surgical Institute of Nanchang University, Nanchang, 330006 China

**Keywords:** Endocrinology, Gastroenterology

## Abstract

The change in gut microbiota is an important mechanism of the amelioration of type 2 diabetes mellitus (T2DM) after bariatric surgery. Here, we observe that the modified jejunoileal bypass effectively decreases body weight gain, fasting blood glucose, and lipids level in serum; additionally, islet β-cell function, glucose tolerance, and insulin resistance were markedly ameliorated. The hypoglycemic effect and the improvement in islet β-cell function depend on the changes in gut microbiota structure. modified jejunoileal bypass increases the abundance of gut *Escherichia coli* and *Ruminococcus gnavus* and the levels of serum glycine, histidine, and glutamine in T2DM rats; and decreases the abundance of *Prevotella copri* and the levels of serum branched chain amino acids, which are significantly related to the improvement of islet β-cell function in T2DM rats. Our results suggest that amino acid metabolism may contribute to the islet β-cell function in T2DM rats after modified jejunoileal bypass and that improving gut microbiota composition is a potential therapeutic strategy for T2DM.

## Introduction

Obesity and type 2 diabetes mellitus (T2DM) are diseases harmful to human health, and their incidence is increasing annually^1^. How to effectively and comprehensively prevent and reverse obesity and T2DM has become a commonly researched topic of global research. Recent studies have shown that metabolic/bariatric surgery (MBS) is the most effective method for the treatment of T2DM^2, 3^. We designed a new MBS procedure called side-to-side jejunoileal bypass plus proximal loop ligation (SSJIBL), which has the advantages of simple operation and recoverability^4^. It has been reported that double-dual jejunoileal bypass which had similar characteristics in some physiological aspects to the SSJIBL has been used in the treatment of obesity and T2DM, and satisfactory results have been obtained^5, 6^.

However, the mechanism of SSJIBL in the treatment of T2DM is unclear. It has been reported that gut microbiota is closely related to the occurrence and treatment of T2DM, and the imbalance of gut microbiota is an important factor in the rapid development of insulin resistance in T2DM^7^. In addition, studies have shown that changing gut microbiota is also an important mechanism of weight loss and glucose-lowering after MBS^8, 9^. Gut microbiota may affect host metabolism by regulating metabolites such as lipopolysaccharides (LPS), bile acids, short chain fatty acids, and amino acids^10^. However, the detailed mechanism of gut microbiota involved in the regulation of energy metabolism and glucose metabolism after SSJIBL requires further research.

In this study, we have observed that SSJIBL effectively reduced body weight gain and blood lipid levels and significantly improved glucose tolerance and islet β-cell function of T2DM rats (Goto-Kakizaki [GK] rats) in a time-dependent manner. Using 16S ribosomal DNA (16S rDNA)-based microbiota analysis and metabonomic analysis, we proved that SSJIBL changed the gut microbiota composition and amino acid metabolism of T2DM rats. SSJIBL may regulate amino acid metabolism by upregulating the function of intestinal membrane transporters, especially ABC transporters. Our study also showed that the abundance of gut *Escherichia coli* and *Ruminococcus gnavus*, and serum glycine increased significantly after SSJIBL, accompanied by a significant decrease in the abundance of gut *Prevotella copri* and branched chain amino acids’ (BCAAs) levels, which has been significantly related to improvement in glucose metabolism.

These results suggest that SSJIBL improves glucose metabolism in a time-dependent manner based on slow changes in the gut microbiome. SSJIBL may improve the islet β-cell function of diabetic rats by changing gut microbiota and regulating amino acid metabolism. Further exploration of the regulatory relationship between gut microbiota, amino acid metabolism and islet β cell function will provide new targets for the treatment of T2DM and metabolic syndrome.

## Results

### SSJIBL reduces body weight gain, food intake, and fasting blood glucose and improves glucose tolerance and insulin sensitivity in GK rats in a time-dependent manner

**Figure 1:**
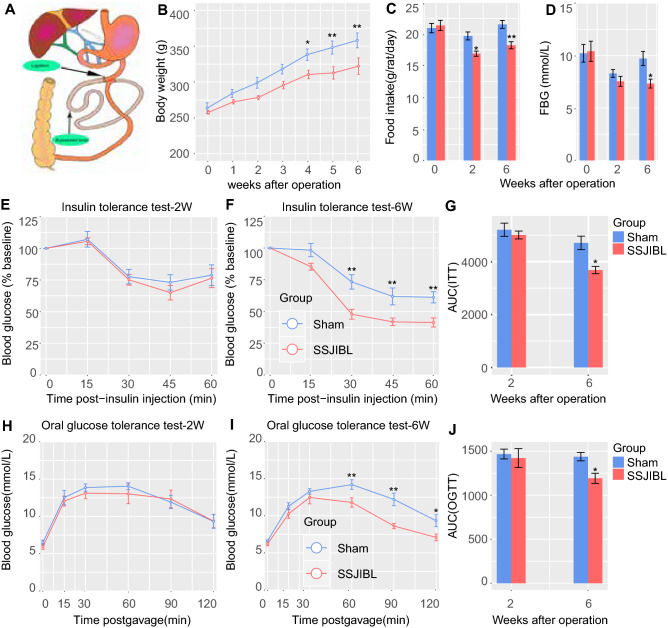
SSJIBL reduced weight gain, food intake, and fasting blood glucose level, and improved glucose tolerance and insulin sensitivity in GK rats in a time-dependent manner. Rats were randomly divided into 2 groups (n = 8 for SSJIBL, n = 7 for Sham). (**A**) For the SSJIBL surgery, a point 40 cm proximal to the ileocecal valve was used as the reference point. Starting proximally from this point to 6 cm distal to the Treitz ligament, approximately 60% of the length of the entire small bowel was bypassed, and bowel continuity was restored by a side-to-side anastomosis between the proximal jejunum and the ileum. The luminal occlusion was performed at the first portion of the bypassed segment by using a ligation of 0 silk suture. (**B**) Body weight of SSJIBL and Sham treated rats. (**C**) Average daily food intake for the above 2 groups of rats 2 W and 6 W postoperation. (**D**) Fasting blood glucose level (FBG). (**E**,**F**) Effect of SSJIBL on percentage of initial blood glucose level during insulin tolerance test (ITT). (**G**) Area under the curve (AUC). (**H**,**I**) Effect of SSJIBL on glucose tolerance measured by oral glucose tolerance test (OGTT). (**J**) AUC. Error bars are expressed as means ± SEM. Statistical significance was determined by two-tailed Student’s t test or two-way ANOVA. *P < 0.05, **P < 0.01.

Eight-week-old GK rats were randomly divided into one operation group (SSJIBL) and one operation control group (Sham). There was no significant difference in body weight between the 2 groups at 1, 2, and 3 weeks after operation; but at 4, 5, and 6 weeks after operation, the body weight of SSJIBL rats was significantly lower than that of Sham rats (Fig. 1B). The food intake of rats in the SSJIBL group decreased 2 weeks after operation, and the difference was more obvious 6 weeks after operation (Fig. 1C). At 2 weeks after operation, there was no significant difference in FBG between the 2 groups; but at 6 weeks after operation, the FBG in the SSJIBL group was significantly lower than that in the Sham group (Fig. 1D). Two weeks after operation, there was no significant difference in the insulin tolerance test (ITT; Fig. 1E,G) and OGTT (Fig. 1H,J) between the 2 groups; 6 weeks after operation, the ITT (Fig. 1F,G) and OGTT (Fig. 1I,J) of rats in the SSJIBL group were significantly better than those in the Sham group.

### SSJIBL improves lipid homeostasis and liver function, promotes the release of incretin hormones, and stimulates the proliferation of pancreatic islet cells

**Figure 2:**
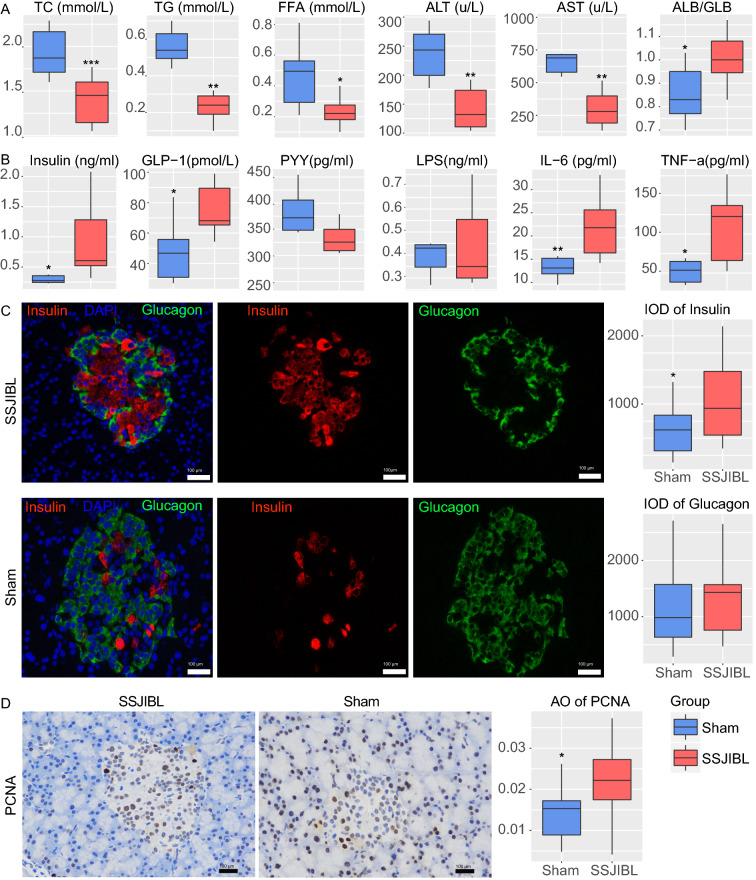
SSJIBL improves lipid homeostasis and liver function, promotes the release of hormones such as insulin and glucagon-like peptid-1, and stimulates the proliferation of pancreatic islet cells. (**A**) Total TC, TG, FFA, ALT, AST, and ALB/GLB levels in blood. (**B**) Insulin, GLP-1, PYY, LPS, IL-6, and TNF-α levels in blood. (**C**) Representative pictures of immunofluorescence of pancreatic tissue (scale bar, 100 μm). Right, integrated optical density (**D**). Representative pictures of immunohistochemistry of pancreatic tissue (scale bar, 100 μm). Right, average optical density (AO). Error bars are expressed as means ± SEM. Statistical significance was determined by two-tailed Student’s t test. *P < 0.05, **P < 0.01, ***P < 0.001.

Six weeks after operation, serum TC, TG, and FFA decreased significantly in SSJIBL-treated rats (Fig. 2A). SSJIBL also had a protective effect on liver function, showing a significant decrease in ALT and AST and a significant increase in the ALB/GLB ratio (Fig. 2A). In addition, SSJIBL increased fasting serum insulin and GLP-1 levels in the SSJIBL group rats, but there was no significant difference in serum PYY levels between the 2 groups (Fig. 2B). Surprisingly, we observed that the levels of serum TNF- α and IL-6 in rats after SSJIBL were higher than those in the Sham group, whereas there was no significant difference in serum LPS level between the 2 groups (Fig. 2B).

We further evaluated the effect of SSJIBL on islet β-cell proliferation in GK rats. Six weeks after operation, the results of immunofluorescence of pancreatic tissue showed that the positive expression of insulin in the islets of SSJIBL-treated rats was significantly higher than that of the Sham group, but there was no difference in the positive expression of glucagon between the 2 groups (Fig. 2C). In addition, immunohistochemistry showed that the expression of islet proliferating antigen (PCNA) was significantly increased in GK rats after SSJIBL (Fig. 2D).

### SSJIBL altered the gut microbiota composition of GK rats in a time-dependent manner

Gut microbiota plays an important role in the occurrence and development of T2DM, and modulation of microbiome is a potential method to prevent and treat T2DM. By performing a pyrophosphate sequencing-based analysis of bacterial 16S rDNA in feces, we examined the effect of SSJIBL on the composition of gut microbiota in GK rats. According to the number of operational taxonomic unit (OTU) information, the OTU of each group was calculated (not considering the abundance of OTU, only considering whether there is OTU or not). The common and unique OTUs between groups are shown by a Venn diagram. The total number of OTU in the SSJIBL group was higher than that in the Sham group at 2 and 6 weeks after operation, and the total number of OTU in the SSJIBL group at 6 weeks after operation was higher than that at 2 weeks after operation.

**Figure 3:**
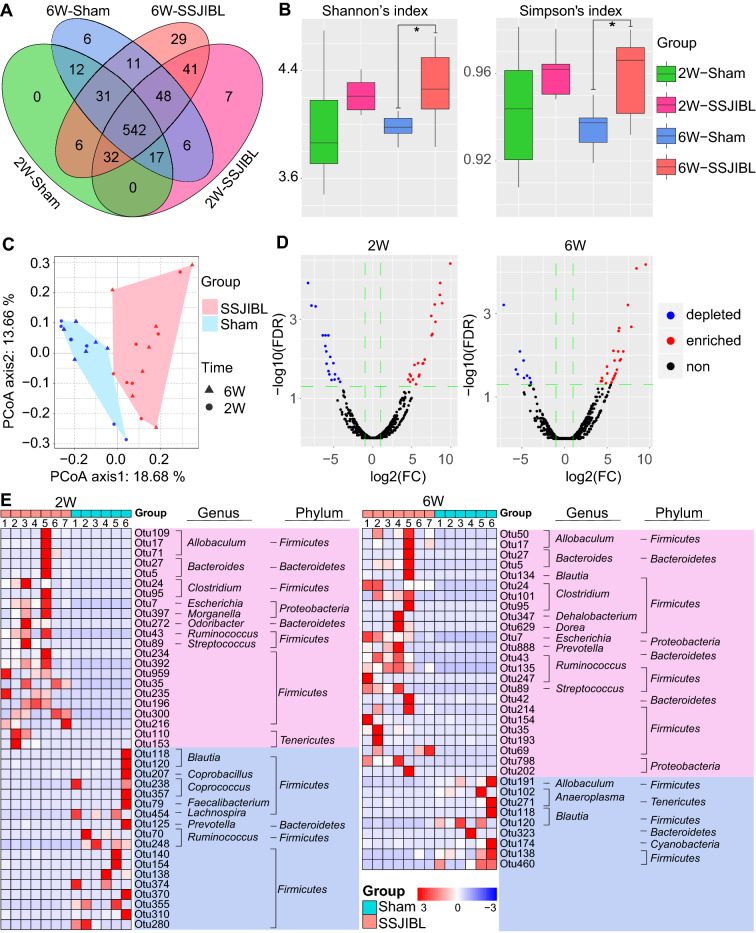
SSJIBL altered the gut microbiota composition of GK rats in a time-dependent manner. (**A**) Venn map. (**B**) α diversity of gut microbiota. (**C**) Bray curtis PCoA analysis of gut microbiota based on the OTU data of Sham and SSJIBL groups. (**D**) Volcano map of different OTUs. (**E**) Heatmap of the different OTUs altered by SSJIBL treatment. The color of the spots in the left panel represents the relative abundance of the OTU in each group. The phylum, and genus names of the OTUs are shown on the right panel. The analyses were conducted using R software version 3.6.2.

In the next step, we analyzed the α diversity of OTU. Because of the high diversity of microbial communities, we used "Shannon’s index" and "Simpson’s index" to evaluate the richness and evenness of α diversity. The α diversity of gut microbiome changed over time after SSJIBL (Fig. 3A). Two weeks after operation, the Shannon index and Simpson index in the SSJIBL group increased, but the difference was not statistically significant (Fig. 3B); 6 weeks after operation, Shannon’s index and Simpson’s index in the SSJIBL group increased significantly. In addition, we used principal coordinate analysis (PCoA) based on the Bray–Curtis distance and observed significant clustering of gut microbiota in the SSJIBL and Sham groups (Fig. 3C).

Furthermore, univariate analysis in the edgeR package was used to identify the specific bacterial phylotypes changed by SSJIBL. The filtering criteria for different OTUs are as follows: The foldchange is greater than or equal to 2, and FDR is less than or equal to 0.05 (Fig. 3D). The results showed 40 changed OTUs (22 up-regulated and 18 down-regulated) 2 weeks after operation and 33 changed OTUs (24 up-regulated and 9 down-regulated) 6 weeks after operation. Phylum-level analysis revealed that most of these altered OTUs belonged to *Firmicutes* and that a small part was from *Proteobacteria* and *Bacteroidetes*. Genus-level analysis showed that the changed OTUs were mainly, for example, *Anaeroplasma*, *Bacteroides*, *Blautia*, *Clostridium*, *Dehalobacterium*, *Dorea*, *Escherichia*, *Prevotella*, and *Ruminococcus* (Fig. 3E).

### SSJIBL up-regulated gut *Escherichia* and *Ruminococcus* abundance and down-regulated *Prevotella* abundance in GK rats

**Figure 4:**
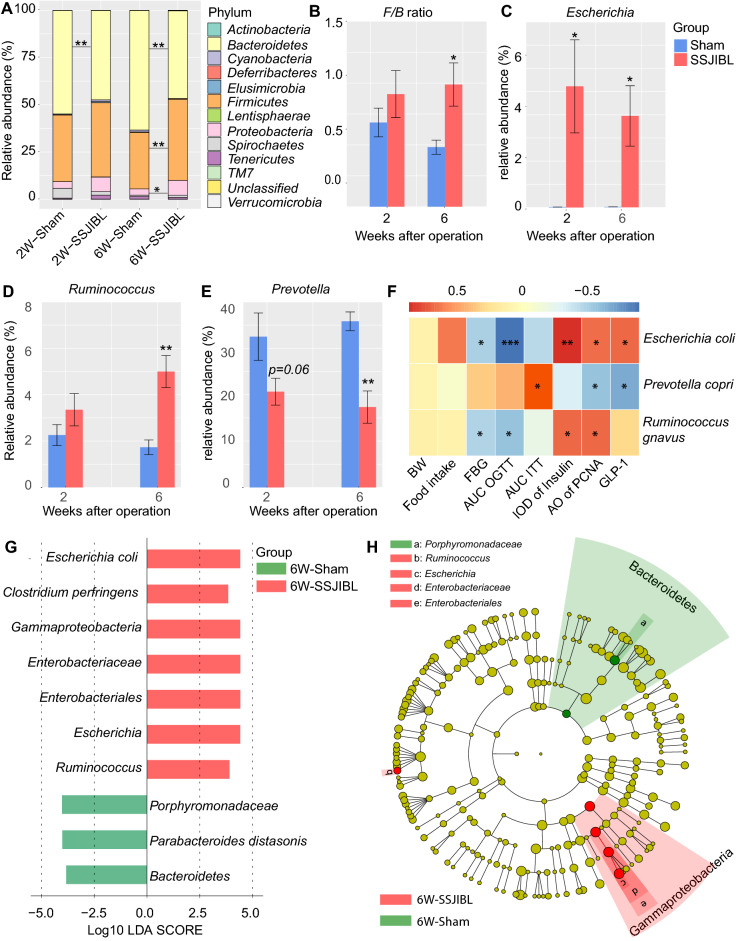
SSJIBL up-regulated gut *Escherichia* and *Ruminococcus* and down-regulated *Prevotella* in GK rats. (**A**) Bacterial taxonomic profiling at the phylum level of intestinal bacteria from two groups 2 weeks and 6 weeks postoperation. (**B**) *Firmicutes*-to-*Bacteroidetes* ratio in the indicated groups. (**C**–**E**) Bacterial taxonomic profiling at the genus level of intestinal bacteria from two groups 2 weeks and 6 weeks postoperation. (**F**) Heatmap analysis of the Pearson correlation of *Escherichia coli*, *Ruminococcus gnavus* and *Prevotella copri* and glucose homeostasis-related indexes. Red represents positive correlation, and blue indicates negative correlation. (**G**) A linear discriminant effect size (LEfSe) analysis was performed (alpha value ≥ 0.05, logarithmic LDA score threshold ≥ 3.0) on two groups together. (**H**) The cladogram represents the phylogenetic relationship of significant OTUs associated with each group. Error bars are expressed as means ± SEM. Statistical significance was determined by two-tailed Student’s t test. *P < 0.05, **P < 0.01, and ***P < 0.001.

To evaluate the overall composition of intestinal bacterial communities in rats in different intervention groups, we analyzed the similarity of bacterial taxonomy at the phylum level. Two weeks after operation, the abundance of *Bacteroides* and *Firmicutes* in feces of rats in the SSJIBL group increased, but there was no significant difference. Six weeks after operation, the abundance of *Firmicutes* and *Proteobacteria* in feces of rats in the SSJIBL group increased significantly, the abundance of *Bacteroides* decreased (Fig. 4A), and the *Firmicutes*-to-*Bacteroides* ratio increased significantly (Fig. 4B).

We further analyzed the taxonomic similarity of bacteria at the genus level. Two weeks after operation, the abundance of *Escherichia* in the feces of rats in the SSJIBL group increased significantly, and the abundance of *Prevotella* decreased (*P* = 0.06). Six weeks after operation, the abundance of *Escherichia* and *Ruminococcus* in the feces of rats in the SSJIBL group increased, and the abundance of *Prevotella* decreased (Fig. 4C–E). Although we observed an increase of *Escherichia* abundance at 2 and 6 weeks after operation, further Linear discriminant analysis Effect Size (LEfSe) showed no significant difference at 2 weeks after operation; at 6 weeks after operation, however, the abundance of *Escherichia* and *Ruminococcus* in feces of rats in the SSJIBL group significantly increased, and the abundance of *Porphyromonadaceae* decreased (Fig. 4G,H).

We then conducted Spearman's correlation analysis between the representative altered specific bacterial phylotypes and the metabolic characterization. The analysis showed that *Escherichia coli* was positively correlated with the level of GLP-1 in serum and the positive expression rate of insulin and PCNA in islets, and negatively correlated with FBG and AUC_OGTT_. There was a positive correlation between *Prevotella copri* and AUC_ITT_, and a significant negative correlation with the positive expression of islet PCNA and the level of serum GLP-1. *Ruminococcus gnavus* was negatively correlated with FBG and AUC_OGTT_, and positively correlated with islet PCNA expression (Fig. 4F).

### Predictive function analysis

**Figure 5:**
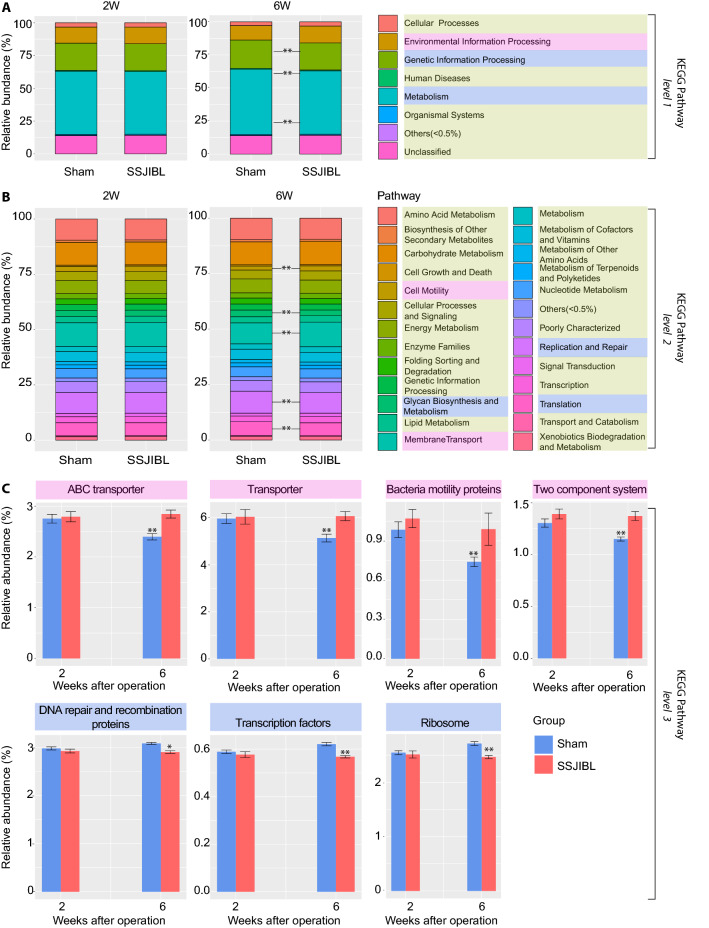
Predicted KEGG functional pathway differences between SSJIBL and Sham groups 2 weeks and 6 weeks postoperation. PICRUSt was used to predict functional potential of microbiomes using 16S rDNA gene sequence data. (**A**) KEGG functional pathway differences at level 1. (**B**) KEGG functional pathway differences at level 2. (**C**) KEGG functional pathway differences at level 3. Error bars are expressed as means ± SEM. Statistical significance was determined by two-tailed Student’s t test. *P < 0.05, **P < 0.01.

Next, to study the potential function of gut microbiome, the relative abundance of the KEGG pathway was predicted by phylogenetic investigation of communities by reconstruction of unobserved states (PICRUSt). Notably, there was no significant difference in predicted gene content in KEGG pathways between the SSJIBL group and Sham group at 2 weeks after operation. At 6 weeks after operation, however, the difference was significant. First-level analysis revealed that "Environmental information processing" was increased, whereas "Genetic information processing" and "Metabolism" was decreased in the SSJIBL-treated group (Fig. 5A). Second-level analysis showed that "Cell motility" and "Membrane transport" were increased in the SSJIBL group, whereas "Glycan biosynthesis and metabolism," "Replication and repair," and "Translation" were decreased significantly in the SSJIBL group (Fig. 5B). Third-level analysis revealed that "ABC transporter," "Transporter," "Bacteria motility proteins," and "Two component system" were significantly higher in the SSJIBL group than in the Sham group (Fig. 5C). However, "DNA repair and recombination proteins," "Transcription factors" and "Ribosome" were decreased in the SSJIBL group.

### SSJIBL alters amino acids metabolism in GK rats

**Figure 6:**
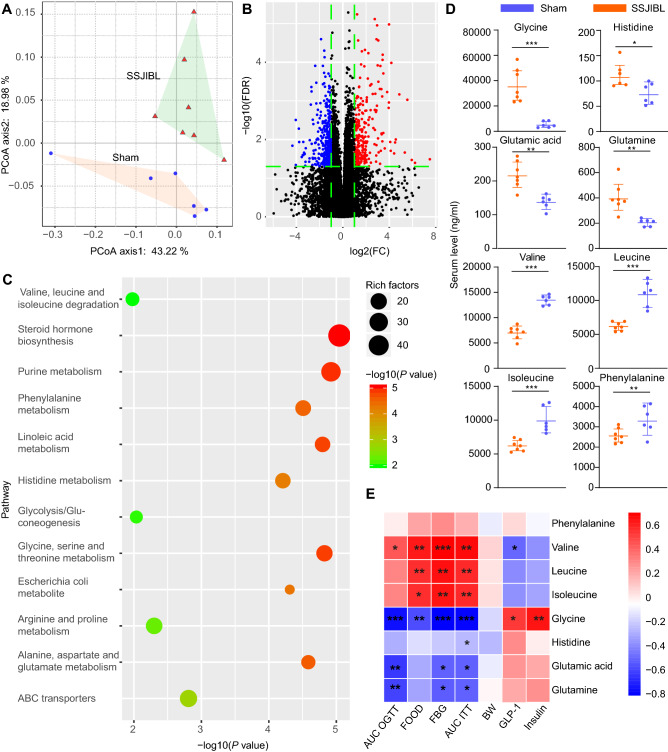
SSJIBL alters amino acids metabolism in GK rats 6 weeks postoperation. (**A**) PCoA analysis score plots for discriminating the fecal metabolome from SSJIBL and Sham groups. (**B**) Volcano map of different metabolites. (**C**) Disturbed metabolic pathways in the SSJIBL versus Sham groups. (**D**) Comparison of circulating levels of glycine, histidine, glutamic acid, glutamine, valine, leucine, isoleucine, and phenylalanine in serum by GC–MS in the indicated groups. (**E**) Heatmap analysis of the Pearson correlation of serum amino acids and glucose homeostasis-related indexes. Red represents positive correlation, and blue indicates negative correlation. Error bars are expressed as means ± SEM. Statistical significance was determined by two-tailed Student’s t test. *P < 0.05, **P < 0.01, ***P < 0.001.

To evaluate the metabolic changes in gut microbiome remodeled by SSJIBL, the serum samples of GK rats were analyzed by ultra-high performance liquid chromatography-quadrupole time-of-flight mass spectrometry (UPLC-QTOF/MS). A total of 16,435 ions were detected in serum. PCoA showed an obvious aggregation of metabolites between the SSJIBL and Sham rats (Fig. 6A). After the intervention of SSJIBL, a wide range of metabolites were altered. Compared with the Sham group, 958 and 1467 metabolites were significantly induced and inhibited, respectively (Fig. 6B). The topology diagram generated by MetaboAnalyst 4.0 (www. metaboanalyst. ca) describes the effect of SSJIBL on these reactive metabolites. After SSJIBL intervention, the main metabolic changes were valine, leucine and isoleucine degradation, phenylalanine metabolism, histidine metabolism, glycine, tryptophan and threonine metabolism, arginine and proline metabolism, alanine, aspartic acid and glutamate metabolism, and ABC transporter (Fig. 6C).

Next, to further clarify the regulatory effect of SSJIBL intervention on serum amino acid metabolism in T2DM rats, we used gas chromatography-mass spectrometry (GC–MS) to analyze the levels of amino acids in different groups of serum samples. The results showed that the levels of glycine, histidine, glutamic acid, and glutamine increased significantly, whereas the levels of valine, leucine, isoleucine, and phenylalanine decreased significantly (Fig. 6D). Spearman's correlation analysis showed that these amino acid levels were positively correlated with metabolic characterization, including food intake, FBG, glucose tolerance, insulin resistance, and serum leptin and insulin levels, among which glycine had the strongest correlation (Fig. 6E).

## Discussion

Lifestyle changes and/or dietary interventions are usually insufficient to maintain long-term weight loss and often result in weight regain. With the increasing number of super-obese patients, MBS is also widely used worldwide. At present, Roux-en-Y gastric bypass (RYGB) and sleeve gastrectomy (SG) are the most widely used types of MBS in the world^3^. Jejunoileal bypass (JIB) was a classic MBS and was obsolete by the mid-1980s because of the serious complications mainly induced by blind loop syndrome^11^. SSJIBL is a modified JIB procedure that the authors of this study had previously designed. It has significant effects on weight loss and hypoglycemia^4, 12^.

Our previous studies have shown that the hypoglycemic mechanism of SSJIBL is intricately linked to the improvement of islet β-cell function^4^. However, the specific mechanism is unclear. We speculated that SSJIBL may improve islet β-cell function in the following manners^13^: (1) elevated gastrointestinal hormones such as GLP-1 and PYY, (2) gut microbiota and their metabolites, (3) reduced glucotoxicity and chronic inflammation.

SSJIBL changed the gut microbiota composition in a time-dependent manner by reorganizing the structure of the gastrointestinal tract. LEfSe analysis and PICRUSt function prediction showed that SSJIBL had no significant change in the gut microbiota of T2DM rats at 2 weeks after operation. However, there were significant changes at 6 weeks after operation (Figs. 4H, 5). Notably, SSJIBL also improved glucose tolerance and insulin sensitivity in T2DM rats in a time-dependent manner, which was consistent with intestinal microbiota remodeling (Fig. 1). These results suggest that the weight loss and hypoglycemic effects of SSJIBL depend on the changes in gut microbiota.

Six weeks after operation, the gut microbiota in the feces of T2DM rats was analyzed by 16S rDNA. We observed that the abundance of *Escherichia* and *Ruminococcus* increased significantly after the intervention of SSJIBL. A recent meta-analysis showed that *Escherichia* and *Ruminococcus* significantly increased whereas the *Alistipes*, *Enterococcus*, *Blautia*, and *Dorea* decreased significantly after RYGB or SG^14^. Our results were similar this meta-anlysis. Although studies have demonstrated that the abundance of *Prevotella* increased significantly after RYGB^15, 16^, we observed that the abundance of *Prevotella* in the gut of T2DM rats decreased significantly after SSJIBL. Further correlation analysis suggested that the abundance of *Prevotella copri* was negatively correlated with islet β-cell proliferation in T2DM rats and positively correlated with AUC_ITT_ (Fig. 4F). Some studies have shown that the level of serum BCAAs in patients with insulin resistance is increased, and the mechanism is that *Prevotella copri* induces the expression of genes related to BCAAs’ biosynthesis and reduces the expression of genes related to BCAAs’ degradation^17^. Our results showed that the levels of valine, leucine, and isoleucine in serum of T2DM rats decreased after SSJIBL, whereas insulin sensitivity was significantly improved. Further study is needed to determine if the decrease in gut *Prevotella copri* abundance after SSJIBL improve insulin sensitivity by reducing the level of serum BCAAs.

Glycine, as a non-essential amino acid in the body, can be synthesized by serine in the body. Its level is negatively correlated with T2DM^18^ and obesity^19^. Glycine supplementation can promote insulin secretion and improve glucose tolerance^20^. The increase in plasma glycine level was also observed after MBS^21^. Glycine can improve glucose metabolism through the *N*-methyl-d-aspartate receptor^22^ and inhibit liver glucose output mediated by the dorsal vagus nerve complex^23^. Gut microbiota is rich in genes related to glycine degradation, an important link glycine metabolism regulation^24^. In our study, the gut microbiota changed significantly after SSJIBL for 6 weeks, which was mainly reflected in the functional enhancement of the ABC transporter (Fig. 5). We speculate that SSJIBL may regulate glycine by increasing the abundance of *Escherichia* and *Ruminococcus*, and then improve islet β-cell proliferation, but this requires confirmation by further research.

We also observed that the levels of serum glutamate increased significantly after SSJIBL, and negatively correlated with FBG and oral glucose tolerance in GK rats. Although the mechanism of glutamate improving glucose tolerance is not very explicit, clinical^25^ and in vitro experiments^26^ have shown that glutamate can stimulate intestinal L cells to secrete GLP-1. The levels of GLP-1^27^ and PYY^28^ increased rapidly after RYGB and SG. GLP-1 and PYY promoted insulin synthesis and secretion by binding to their receptors on islet β cells^29^, which could promote islet β-cell proliferation and inhibit its apoptosis^30^. We observed that the fasting serum GLP-1 level in SSJIBL was significantly higher than that in the Sham group (Fig. 2A). The elevated level of glutamate after SSJIBL may stimulate intestinal endocrine cells to secrete GLP-1 and then promote islet β-cell proliferation, but the detailed mechanism of action requires further confirmation.

A notable phenomenon is that the levels of serum lipids decreased in T2DM rats after SSJIBL, whereas the serum inflammatory factors IL-6 and TNF-α increased (Fig. 2A). Inflammatory factors can the damage viability of islet β-cell and cause insulin resistance, which is an important mechanism in the pathogenesis of diabetes^31, 32^. Some studies have reported lower levels of pro inflammatory cytokines in patients receiving RYGB^33^ and LSG^34^, which are closely related to the glucose-lowering and the improvement in insulin released by islet β-cells. However, some studies have revealed that the expression of IL-6, IL-8, and TNF-α genes in visceral adipose tissue increased significantly after RYGB^35^. Recently, Rakotoarivelo Volatiana et al. observed no significant difference in the expression of inflammatory factors such as IL6, IL-1 β, and TNF-α between obese patients with (or without) T2DM and non-obese patients^36^. Together, we speculate that the mechanism by which SSJIBL improves islet β-cell function is not by reducing chronic inflammation.

In summary, SSJIBL could improve islet β-cell function by regulating amino acid metabolism through modulation of gut microbiota. The changes in gut microbiota of rats by SSJIBL mainly included the enrichment of *Escherichia coli* and the decrease of *Prevotella copri*. *Escherichia coli* may be a potentially beneficial intestinal bacterium that improves glucose tolerance and more researches should be undertaken in depth. Our findings are valuable for considering changes in gut microbiota as a new mechanism for MBS weight loss and hypoglycemia.

## Materials and methods

### Animals

Animal experiments were conducted according to the Animal experiment Guide of Nanchang University and approved by the Animal Ethics Committee of Nanchang University. Eight-week-old GK male rats (261 ± 10.1 g) were provided by Slac Laboratory Animal Co. Ltd (Shanghai, China). The rats were kept in individually ventilated cages. Before the experiment, they adapted to the environment for at least 1 week and were free to use tap water and standard rat feed. The rats were randomly divided into the SSJIBL group and Sham operation group. The body weight was recorded every week after operation. Food intake, fasting blood glucose (FBG), oral glucose tolerance test (OGTT), and insulin tolerance test (ITT) were recorded at 2 and 6 weeks after operation.

### Side-to-side jejunoileal bypass plus proximal loop ligation (SSJIBL)

After fasting for 14 h, an operation was performed on the rats while they were under anesthesia (isoflurane, 4% for induction and 2% for maintenance). The abdomen was shaved, and the peritoneal cavity was accessed through a 4-cm midline incision.

For the SSJIBL group, a point 40 cm proximal to the ileocecal valve was used as the reference point. Starting proximally from this point to 6 cm distal to the Treitz ligament, approximately 60% of the length of the entire small bowel was bypassed, and bowel continuity was restored by a side-to-side anastomosis between the proximal jejunum and the ileum. The luminal occlusion was performed at the first portion of the bypassed segment by using a ligation of 0 silk suture^4^.

For the Sham group, the peritoneal cavity was accessed through a 4-cm midline incision, and the intestine was gently manipulated. The abdominal cavity was closed using 3–0 silk suture. The duration of the surgical procedure was approximately 45 min.

All rats were given 10 ml of sterile saline subcutaneously after surgery and were housed in individual cages to recover from anesthesia.

### OGTT and ITT

OGTT and ITT were performed 2 and 6 weeks after operation. OGTT was performed in rats after fasting for 14 h. At the end of fasting, baseline blood glucose readings were obtained from the tail. GK rats were fed with 20% glucose (1 g/kg) intragastrically. Blood glucose was measured at 0, 15, 30, 60, 90, and 120 min, respectively, and the area under the glucose tolerance curve (AUC_OGTT_) was calculated. ITT was performed in GK rats after fasting them for 6 h. After the baseline blood glucose reading, the rats were injected intraperitoneally with insulin (0.5 IU/kg), and the blood glucose levels were measured at 0, 15, 30, 45, and 60 min, respectively. Insulin sensitivity was evaluated by the ratio of blood glucose to basal blood glucose at each time point, and the area under the insulin tolerance curve (AUC_ITT_) was calculated.

### Biochemical tests

Glucose levels were measured by an electronic glucometer (Accu-Chek Performa, Roche Diagnostic, Switzerland) on blood obtained through the tail vein of conscious rats. For the FBG, the food was taken away at 8:00 a.m., after 8 h of fasting, the glucose level was measured at 8:00 p.m. before surgery and at 2 and 6 weeks postoperation.

After fasting for 1 night, the rats were killed, and the blood was taken from the portal vein to the biochemical tube with coagulant. After 15 min of 3000 rpm centrifugation at 4 °C, the isolated serum was immediately transferred to a new test tube and stored in – 80 °C until analysis. Serum total cholesterol (CH), triglyceride (TG), free fatty acid (FFA), alanine aminotransferase (ALT), aspartate aminotransferase (AST), and albumin/globulin (ALB/GLB) ratio were measured by automatic biochemical analyzer. The analysis and test were conducted by the biochemical laboratory of the second affiliated hospital of Nanchang University.

Serum insulin, glucagon-like peptide-1 (GLP-1), peptide YY (PYY), lipopolysaccharide (LPS), interleukin-6 (IL-6), and tumor necrosis factor α (TNF-α) levels were measured on blood obtained through the portal vein. Blood samples were immediately centrifuged at 3000 rpm for 13 min. Serum was removed immediately and stored at − 80 °C until analysis. All serum indexes were detected by enzyme-linked immunosorbent assay kit (Merck Millipore, USA).

### Immunohistochemical analysis

Six weeks after operation, the pancreas of rats was fixed with 4% formalin, embedded in paraffin, and sectioned at 5 mm. After dewaxing, hydration, and rinsing, 3% hydrogen peroxide was added to block the endogenous peroxidase. Next the slice was closed with 3% BSA for 30 min and incubated overnight with 1:1000 primary antibody (Servicebio, Wuhan, China) at 4 °C. Then, the second antibody (HRP labeled goat anti-rabbit, Servicebio, Wuhan, China) was added and incubated for 50 min. Finally, DAB was added to develop color and counterstain. Four slices were taken from the specimen, and 3 high power fields (200×) were randomly selected from each slice. The area and integral optical density (IOD) were measured by image analysis software IPP (Image-Pro Plus 6.0). The average optical density (AO), was calculated by dividing the IOD by the islet pixel area, and the AO was statistically analyzed.

### Immunofluorescence

After the slices were dewaxed to water, the tissue was covered with protease K working solution and incubated in a 37 °C incubator. A proper amount of reagent 1 (TdT) and reagent 2 (dUTP) in a TUNEL kit (Roche, Germany) were mixed at 1:9, added to the covered tissue in the circle, and incubated in an incubator at 37 °C for 2 h. The nuclei were re-stained with DAPI staining solution. The sections were observed under a fluorescence microscope (NIKON ECLIPSEC1, Japan), and the images were collected. Each slice in each group was randomly selected with at least three 200-fold visual fields to be photographed. We used Image-Pro Plus 6.0 software to convert red fluorescent monochromatic photos into black-and-white pictures; next, we selected the same black as the unified standard to judge the positive of all photos and analyze each photo to obtain the cumulative optical density value (IOD) of each photo.

### 16S rDNA amplicon sequencing

According to the manufacturer's instructions, MagPure fecal DNA KF kit B (Magen, China) was used to extract microbial community DNA. Using Qubit dsDNA BR Analysis Kit (Invitgen, USA), DNA was quantified with Qubit fluorophotometer, and Aliquot was run on 1% agarose gel for a quality check. After DNA was extracted, 1 μg genomic DNA, was randomly cut with Covaris and purified by an AxyPrep Mag PCR purification kit. The average size of DNA selected by Agencourt AMPure XP medium kit was 200 ~ 400 bp. The end of the fragment was repaired with the end repair mixture and then purified. The repaired DNA was combined with the mixture of A-Tail; next, the Illumina joint was connected to the adenosine 3′-terminal DNA and then purified. The product was selected according to the size of the insert. Several rounds of PCR amplification were conducted with PCR primer cocktail and PCR Master Mix to enrich the DNA fragment connected by Adapter. After purification, the library was identified by Agilent 2100 biological analyzer (Agilent) and the ABI StepOnePlus Realtime PCR system. Finally, 16S rDNA gene sequence tags, corresponding to the hypervariable V4 region, were generated using the HiSeq2500 sequencing platform (BGI, Shenzhen, China).

### Untargeted metabolomics profiling of serum

Untargeted metabolomics profiling was performed by the Beijing Genomics Institute (BGI) as described^37^. The serum samples were stored at – 20 °C or 30 min and then melted in the refrigerator at 4 °C. Each sample (including QC) 40 µL was added to the corresponding 96-well plate; methanol 120 μL was added to the new EP tube with 40 μL sample (including QC); the film was sealed and vibrated for 1 min, placed in the refrigerator at − 20 °C for 30 min, and centrifuged at 4 °C with 4000 rpm for 20 min. We removed the 96-well plate from the centrifuge, put 20 μL of each hole in the new 96-well plate, added 180 μL 50% methanol to dilute, mixed, put 20 μL of each hole in the sample tank and mixed that into the QC sample of the machine, and put 80 μL of each hole in the new 96-well plate; next, we sealed the 96-well plate with a heat sealing instrument, simultaneously sealed the film with the protein precipitated plate, and stored it at − 80 °C.

The treated serum was separated by liquid chromatography on ACQUITY UPLC BEHC18 (100 mm × 2.1 mm, 1.7 μm) by using the 2777C UPLC system and analyzed by mass spectrometry on the SYNAPT G2 XS quadrupole time-of-flight system (all equipment was from Waters technology company, USA). The liquid phase parameters were as follows: mobile phase A: water, mobile phase B: acetonitrile; gradient elution procedure: 0 ~ 1 min, 99% A-70% A; 1 ~ 10 min, 70% A-20% A; 10 ~ 10.1 min, 20% A-5% A; 10.1 ~ 11 min, 5% A; 11 ~ 11.1 min, 5% A-99% A; 11.1 ~ 12 min, 99% A; flow rate: 0.5 mL/min; and injection volume: 10 μL.

The working parameters of the mass spectrometer are set to (capillary, 2 kV; sampling cone, 40 V; source temperature, 110 °C; Desolvation temperature, 350 °C; Desolvation/cone gas, 80/50 [L/h]; source offset, 80).

Peak extraction and identification are mainly realized by commercial software Progenesis QI (version 2.2, Waters technology), including peak alignment, peak extraction, normalization, deconvolution, and ion identification.

HPLC grade acetonitrile, methanol, and formic acid were purchased from Merck (Darmstadt, Germany). Ultra-pure water is purified by Milli-Q academic Water purification system (Millipore, Bedford, MA, USA). Other reagents used are at least analytical grade.

### Targeted amino acid quantification

Targeted amino acid quantification was performed by the Beijing Genomics Institute (BGI) as described^38^. Twenty amino acids (glycine, alanine, serine, proline, valine, threonine, cysteine, isoleucine, leucine, asparagine, aspartic acid, lysine, glutamine, glutamic acid, methionine, histidine, phenylalanine, arginine, tyrosine, tryptophan) were quantitatively analyzed by UPLC-Q Orbitrap high resolution mass spectrometry. The chromatographic column was Hypersil GOLD (100 × 2.1 mm × 1.9 µm, Thermo Fisher Scientific). Additionally, 20.0 µl serum was precipitated with 100 µl ice acetonitrile, mixed with eddy current for 5.0 min, and centrifuged at 13,000*g* at 4 °C for 10.0 min. The supernatant (80.0 µl) was transferred to a clean injection vial. On the Thermo Hypersil GOLD column, the 20 mM ammonium formate aqueous solution (adjusted by formic acid to pH to 3.0)/0.2% formic acid acetonitrile was used as the mobile phase, and the 20.0 µl test sample was injected at the flow rate of 0.3 mL/min. The ion source is electrospray ionization source (ESI), with positive and negative ion switching scanning. Q Orbitrap high resolution mass spectrometry detector is used in PRM mode. The parameters of the chromatographic system, ion source, and mass spectrometry detector are optimized to obtain the required sensitivity. The data acquisition time is set to 7.0 min.

### PICRUSt analyses and KEGG pathway enrichment analyses

We use the software PICRUSt^39^ to predict the possible levels of KEGG pathways (www.kegg.jp/kegg/kegg1.html)^40–42^ and abundance values as well as COG function information and abundance values in 16 s sequencing samples. The Fig. 5 shows the KOs/COGs difference statistics (Supplementary Information).

### Ethics approval

All animal studies were approved by the laboratory Animal Ethics Committee of Nanchang University. Standard animal care and laboratory guidelines were followed according to the ARRIVE guidelines.

### Statistical analysis

Data are expressed as the means ± SEM. All analyses were performed using GraphPad Prism version 8.0 and the level of significance was set at 0.05. The area under the curve (AUC) was calculated using trapezoidal integration. The differences between two groups were analyzed by Student’s *t* test. Body weight, food intake changes, FBG, OGTT and ITT over time were analyzed using two-way analysis of variance (ANOVA). The Bonferroni test was performed for pair-wise comparisons between groups. Differential metabolites were defined as those with variable importance in the projection (VIP) > 1.0 obtained from OPLS-DA and adjusted *P* values less than 0.05. Statistical significance is represented as follows: *P < 0.05, **P < 0.01, ***P < 0.001.

## Supplementary Information

Supplementary Information.
