# Supplementary material for: The role of gut microbiota and amino metabolism in the effects of improvement of islet β-cell function after modified jejunoileal bypass
Source: Sci Rep. 2021 Feb 26;11:4809. doi: 10.1038/s41598-021-84355-x (PMC7910448; doi:10.1038/s41598-021-84355-x)
Supplement: Supplementary file 1 — Supplementary Information. [file 41598_2021_84355_MOESM1_ESM.docx]

***Title page***

**The role of gut microbiota and amino metabolism in the effects of improvement of islet β-cell function after modified jejunoileal bypass**

**Short Title: Modified jejunoileal bypass: metabolic effects**

Cai Tan^1^, Zhihua Zheng ^2^, Xiaogang Wan ^2^, Jiaqing Cao ^2^, Ran Wei^3^, Jinyuan Duan ^4*^

**Affiliations:** ^1^Department of women’s health, Maternal and child health hospital of Jiangxi province, Nanchang (330006), China, ^2^Department of General Surgery, Department of Gastrointestinal Surgery, the Second Affiliated Hospital of Nanchang University, Nanchang (330006), China, ^3^Department of Colorectal Surgery, National Cancer Center/National Clinical Research Center for Cancer/Cancer Hospital, Chinese Academy of Medical Sciences, Peking Union Medical College, Beijing (100021), China. ^4^Department of General Surgery, the First Affiliated Hospital of Nanchang University, Gastrointestinal Surgical Institute of Nanchang University, Nanchang (330006), China.

**SUPPLEMENTAL METHORDS**

**Untargeted metabolomics profiling of serum**

The serum samples were stored at -20℃ for 30 minutes and then melted in the refrigerator at 4℃. Each sample (including QC) 40 uL was added to the corresponding 96-well plate; methanol 120 μL was added to the new EP tube with 40 μL sample (including QC); the film was sealed and vibrated for 1 min, placed in the refrigerator at -20℃ for 30 min, and centrifuged at 4℃ with 4000 rpm for 20 min. We removed the 96-well plate from the centrifuge, put 20 μL of each hole in the new 96-well plate, added 180 μL 50% methanol to dilute, mixed, put 20 μL of each hole in the sample tank and mixed that into the QC sample of the machine, and put 80 μL of each hole in the new 96-well plate; next, we sealed the 96-well plate with a heat sealing instrument, simultaneously sealed the film with the protein precipitated plate, and stored it at -80℃.

The treated serum was separated by liquid chromatography on ACQUITY UPLC BEHC18 (100mm × 2.1mm, 1.7 μm) by using the 2777C UPLC system and analyzed by mass spectrometry on the SYNAPT G2 XS quadrupole time-of-flight system (all equipment was from Waters technology company, USA). The liquid phase parameters were as follows: mobile phase A: water, mobile phase B: acetonitrile; gradient elution procedure: 0～1 min，99% A-70% A；1~10min，70% A-20% A；10~10.1 min，20% A-5% A；10.1~11 min，5% A；11~11.1 min，5% A-99% A；11.1～12 min，99% A；flow rate: 0.5 mL/min；and injection volume:10 μL.

The working parameters of the mass spectrometer are set to (capillary, 2kV; sampling cone, 40V; source temperature, 110℃; Desolvation temperature, 350℃; Desolvation/cone gas, 80/50 [L/h]; source offset, 80).

Peak extraction and identification are mainly realized by commercial software Progenesis QI (version 2.2, Waters technology), including peak alignment, peak extraction, normalization, deconvolution, and ion identification.

HPLC grade acetonitrile, methanol, and formic acid were purchased from Merck (Darmstadt, Germany). Ultra-pure water is purified by Milli-Q academic Water purification system (Millipore, Bedford, MA, USA). Other reagents used are at least analytical grade.

**Targeted amino acid quantification**

Twenty amino acids (glycine, alanine, serine, proline, valine, threonine, cysteine, isoleucine, leucine, asparagine, aspartic acid, lysine, glutamine, glutamic acid, methionine, histidine, phenylalanine, arginine, tyrosine, tryptophan) were quantitatively analyzed by UPLC-Q Orbitrap high resolution mass spectrometry. The chromatographic column was Hypersil GOLD (100 × 2.1mm × 1.9 um, Thermo Fisher Scientific). Additionally, 20.0 ul serum was precipitated with 100 ul ice acetonitrile, mixed with eddy current for 5.0 mins, and centrifuged at 13000 g at 4°C for 10.0 mins. The supernatant (80.0 ul) was transferred to a clean injection vial. On the Thermo Hypersil GOLD column, the 20 mM ammonium formate aqueous solution (adjusted by formic acid to pH to 3.0)/0.2% formic acid acetonitrile was used as the mobile phase, and the 20.0 ul test sample was injected at the flow rate of 0.3 mL/min. The ion source is electrospray ionization source (ESI), with positive and negative ion switching scanning. Q Orbitrap high resolution mass spectrometry detector is used in PRM mode. The parameters of the chromatographic system, ion source, and mass spectrometry detector are optimized to obtain the required sensitivity. The data acquisition time is set to 7.0 mins.
